# CBI-20: Psychometric Properties for the Coping Behaviors Inventory for Alcohol Abuse in Brazil

**DOI:** 10.3389/fpsyt.2018.00585

**Published:** 2018-11-13

**Authors:** Hilda M. R. M. Constant, Carmen Moret-Tatay, Mariana Canellas Benchaya, Margareth da S. Oliveira, Helena M. T. Barros, Maristela Ferigolo

**Affiliations:** ^1^Federal University of Health Sciences of Porto Alegre, Porto Alegre, Rio Grande do Sul, Brazil; ^2^Universidad Católica de Valencia San Vicente Mártir, Valencia, Spain; ^3^Pontifícia Universidade Católica do Rio Grande do Sul, Porto Alegre, Rio Grande do Sul, Brazil

**Keywords:** coping behaviors, alcoholism, counseling, psychometrics, coping, telehealth, multigroup

## Abstract

For any professional, it is of crucial importance to know not only how coping styles and strategies are present in an individual, but to know about its role to the treatment of alcohol abuse. Moreover, new approaches have emerged in this area in terms of relapse prevention and the counseling by phone can be an alternative. The aim of this study is to examine the factor structure of the Coping Behaviors Inventory (CBI) and to test its invariance across groups face-to-face and phone counseling in Brazil. For this purpose, two studies were carried out: study I, the factor structure was revisited in terms of exploratory factor analysis. Study II, face-to-face and phone counseling were examined through confirmatory factor analysis and multigroup analysis. The results confirmed the 4-factor solution with a revised model for the removal of 16 items. Thus presented, a reduced version with better indexes than the previous versions developed over the last 30 years that was ones reformulated from 60 items. The Internal consistency for study I presented α = 0.90 and homogeneity was between 0.17 and 0.5). In addition the KMO = 0.9 = 0.932, ${\text{X}}_{\left(\text{df}=630\right)}^{2}$ = 6091.94, *p* < 0.0 < 0.001. In study II, cronbach's alpha = 0.91 and homogeneity 0.23–0.61 (telemedicine treatment) and α = 0.90 0.17 to 0.63 (face-to-face treatment). In the CFA, the examination of the current version has better fit than the that the traditional model. Moreover, the new version showed convergent validity with the IDHEA questionnaire. In the multigroup analysis no significant changes between groups to a metric level. Finally, the Brazilian version of inventory showed no differences between the phone counseling and face-to-face participants in a metric level after a multigroup analysis.

## Introduction

Alcohol is one of the most socially acceptable drugs that exist in modern times. However, its consumption is responsible for over 3 million deaths a year worldwide (1). According to recent surveys, alcohol has the highest rate of dependence or abuse (2). It is well known that quitting a drug of abuse and remaining abstinent is a complex process. Not surprisingly, this is a subject of interest for healthcare systems. In this way, we have witnessed a vast number of measures carried out in this field that have aimed to provide better and more effective assistance to the population. As follows, barriers such as distance and time have been addressed in a vast number of ways (3). Phone counseling is one of the options to overcome these barriers that have emerged over the last decade, offering promising results. Its potential to increase the efficiency of healthcare for chronic diseases has been studied, as in the case of alcohol abuse (4, 5). Furthermore, some research has indicated that this tool might provide a way for the patient to not suffer the stigma of being identified as an addict (6, 7). This issue seems to be crucial in countries such as Brazil, not only because of its geographical extension; it is also because the prevalence of alcohol-related disorders affect 4.1%, and 2.3% is dependent on alcohol dependence (8). To such an extent that the traditional relapse rate in alcohol users reaches 44% in the first 12 months (9). Moreover, this rate is even increased for a period of 4 years abstinence, where relapse rates reach almost 90% with no follow-up treatment (10). Other study estimates that in short-term the rates vary between 20 and 50% (11). Therefore, the Relapse Prevention (RP) under the phone counseling modality has been an important tool designed to help this population. The logic behind it is that it aims to identify risk situations and coping strategies related to situations of abuse, increasing contact with the patient. This can be a fundamental aspect in the recovery of any additive behavior.

One of the main reasons to have frequent contact with a patient under treatment is to avoid a relapse. This is a critical moment because one should bear in mind that it is rather a complex process which is directly related to the coping styles and strategies employed in the past (12). In this way, it was suggested that patients who persevere in their behavior might have a higher sensitivity to the stimulating effects of alcohol, and this might be also related to a lower cortisol and sensitivity (13–15). Furthermore, and since coping styles are well-recognized to quit alcohol, studies have examined whether these styles may predict how patients respond to treatment. According to current literature (16, 17), those patients with stronger social support, as well as, active coping strategies, were less likely to relapse. Other supporting evidence have been found in terms of odds ratios, sensitivity and mediational models (18). Previous studies also have shown that individuals who have a higher repertoire of effective strategies also have a greater chance of quitting consumption of psychoactive substances and especially in relation to alcohol (12, 19–21).

Strategies and coping styles, might be defined as behaviors used by individuals to deal with certain situations (22). These strategies are critical in order to quit, and obviously, a determining factor in the success of abstinence (12, 23, 24). The study of coping strategies has been widespread during recent years, with a large body of instruments being developed in other areas with regards to stress and health problems (25–28). However, the literature on the assessment of coping strategies in alcohol abusers is rather scarce for the Brazilian population. Thus, a study sought a cross-cultural adaptation of the traditional tool called the Coping Behaviors Inventory (CBI) which is used only with alcohol users (29). Constructed in England and designed to explore the behaviors and thoughts used by alcoholics to prevent, avoid or control the drinking (20). In its first version of 60 items the first four factors, accounted for 54% of the variance and were thought to adequately summarize the inventory. Already in the reexamination with 36 items the first four factors, which now amounted represented 49% of the variance. At that time the varimax rotation was used without taking into consideration that the items can corelate with each other. Items like 8 and 30 migrated from factor 4 in the original model to factor 3 because they had better saturation in this factor. The authors believed that this would clarifies the factor. In view of its satisfactory results, not surprisingly, an analysis of its factor structure seems recommendable. By virtue of that, emphasize the importance of evaluating the strategies to stop consuming alcohol with a instrument current. A similar study was conducted in Spain that report other structure of the CBI (27). The main advantage of this inventory is it is an easy specific application for alcohol users to quit drinking. Furthermore, the ability to measure change the CBI can be proved in some studies that shown that the test to be a sensitive indicator of change (21, 30, 31). However, there are many questions that remain unclear with regards to its factor structure and it invariance across classical treatment, face-to-face and by phone counseling. There are few studies that investigating on telemedicine in brazilian population. Telephone-based monitoring might be a way a patient talks on the alcoholism more sincerely as this is an anonymous a service. Moreover, it overcomes barriers such as distance. These are potential advantage in a country such Brazil, geographically so diverse. Thus, tools that allow professionals to assess these goals are needed. Therefore, the aim of this study is to examine the CBI factor structure and its invariance across the groups cited. This will shed light on the identification of coping strategies in such a complex process as the abstinence one, providing a needed tool in this profile of the population in Brazil. In this way, the original scale structure for the CBI was revisited, examining its psychometric properties among participants undergoing treatment from phone counseling compared to the traditional face-to-face service.

## Materials and methods

### Participants

The inclusion criteria were as follows: (i) participants over 18 years old, (ii) participants wanted to quit alcohol. Participants who did not agree to participate in the study or did not follow it until the end, were excluded. Two sample selections were carried out, where three groups of participants volunteered to take part. The recruitment was carried when participants contacted the service by phone calls or in the inherent health care system for participants in the face-to-face group. This service offered free and anonymous telephone counseling and aimed promote the cessation of alcohol use and they were asked for voluntary participation. Thus, they must give voluntary informed consent to participate in research as described in the Ethic section. The data recruitment was carried out by 40 research assistants, who were previously selected and trained.

Participants who did not agree to participate in the study or did not follow it until the end, were excluded. Two sample selections were carried out, where three groups of participants volunteered to take part. A first sample (study I) of 670 participants attending a phone counseling service that covers the whole Brazilian population was employed for the exploratory factor analysis or in other words, EFA. The sample was composed of 135 women and 535 men with mean age of 31.46 years, standard deviation 8.43 and an age range from 18 to 60. 31.4% of participants had no studies, 54.3% were high school graduates and 14.3% had attended further or higher education. Here, 79.3% were active workers and 20.7% were unemployed. With regards to their marital status, 32.9 % were married, 12% were divorced, 54.8% were single and 0.3% were widowed. Finally, a total of 7.5% of participants consumed only alcohol, 9.6% alcohol as well as tobacco, and 82.9% alcohol plus other illegal drugs.

Two new different samples (study II), similar to the previous one, volunteered to take part: one undergoing telephone counseling and a second sample receiving face-to-face treatment. Thus, a sample of 221 participants (47 women and 174 men with mean age of 33.57 years, standard deviation 9.62 and age range from 18 to 63) took part in the first group undergoing telephone therapy. 41.9% of participants had no studies, 45.4% were high school graduates and 12.7% had attended further or higher education. Moreover, 76.8% were active workers and 23.2% were unemployed. With regards to their marital status, 31.3% were married, 12.9% were divorced, 54.8% were single and 0.9% were widowed). A second sample, which was recruited face-to-face, consisted of 232 participants (56 women and 175 men with mean age of 39.13 years, standard deviation 10.9 and age range from 18 to 60). 47.6% of participants had no studies, 42.1% were high school graduates and 10.3% had attended further or higher education. Here, 61% were active workers and 39% were unemployed. With regards to their marital status, 28.8% were married, 20.2% were divorced, 48.1% were single and 3% were widowed. The aim of using independent samples was to conduct a confirmatory factor analysis (CFA) for the CBI: one sample with the original characteristics of the face-to-face participants and a second one with the characteristics of interest in telephone counseling. Moreover, as some differences were noticed in terms of psychometric properties across factors, another questionnaire was included. The aim of this action was to test the convergent validity of these changes. This sample follow the similar sociodemographic characteristics that the previous ones.

### Materials

All participants filled in a questionnaire consisting of sociodemographic information and aspects related to patterns of consumption. Therefore, the instruments included were:

- Questionnaire of sociodemographic information: Questions about gender, age, education, marital status, and family income.
- Coping Behaviors Inventory–CBI: Adapted to be applied to the Brazilian population. The inventory has 36 items related to coping strategies to avoid situations of risk for the consumption of alcoholic beverages. This instrument was developed from reports of alcoholic patients who described in detail the methods used to prevent relapses and classifies the coping strategies of 4 factors: F1-positive thinking; F2-negative thinking; F3-distraction/avoidance; and F4- social support. The answer refers to the frequency with which a given strategy is used on the Likert scale: 0 = Usually, 1 = Often, 2 = Sometimes, and 3 = Never. The factor score is obtained by adding up the answers of the total raw score or by dividing the total sum by the number of items to obtain an average. Lower scores mean the strategies were used more frequently.
- Inventário de Habilidades de Enfrentamento Antecipatório para Abstinência de Álcool e Outras Drogas (IDHEA-AD) (32). It is the only specific instrument for existing alcoholics in Brazil. It is an questionnaire of self-report and has 30 items related to a specific situation and a possible behavioral response to the demands of the situation. Therefore, the respondent should indicate one of four possible points of response, estimating how often he responds in the way he describes at that moment in his life: never, not infrequently, often or always. The IDHEA-AD results in a total score, with the sum of the scores of all items, and 3 factor scores, with the sum of the scores of subsets of items. Factor 1-Assertiveness and planning for situations of high risk of consumption of Substances. Factor 2-Emotional expression of positive feelings for the maintenance of abstinence. Factor 3-Emotional self-control in adverse situations.

### Design and data analysis

Firstly, a cross-sectional study was carried out to revisit the internal structure of the CBI in terms of internal consistency and factor solution. Secondly, the 4-factor solution was examined across groups undergoing face to face and phone counseling. In this way, an invariance study was undertaken. The data collections were taken from May 2011 to October 2014 (study I) and July 2015 to July 2016 (study II) from different specialized services for chemical dependence. For the data analysis, the SPSS (Statistical Package for the Social Science, version 23.0) and AMOS 18 were employed.

Descriptive statistics (measures of central tendency and dispersion, frequency distribution and percentage) were used in the characterization of the sample. The assessment of the original instrument structure was carried out by Factorial Analysis in two stages: EFA and CFA were undertaken first of all. In the EFA, the method of principal axis factoring was used in order to investigate the behavior of original items. Here, one should bear in mind that the CBI questionnaire follows a polychorical point scale. This might present some instability on the data, for this reason, a bigger sample size was employed. Assumption of Normality and Continuity also were revisited, even if different authors point out that the violation does not have statistically significant consequences on the results (33, 34). According to Jöreskog (35), the estimates obtained from variables with asymmetric distribution are not severely altered if the values of the root mean square error (RMSEA) index are kept within the accepted standards (0–0.08). As commonly done in the literature, the study also measured the appropriateness of model which occurred through the correlation matrix, Kaiser-Meyer-Olkin (KMO) and Bartlett's test of sphericity. For the rotation of the factors, an orthogonal Varimax rotation was suggested in the literature [also suggested by (27)] (20, 27). However, a Promax option was chosen since the factors correlated and an overlap across them is suggested. In the CFA this statistical analysis is more detailed and accurate when compared to EFA, directly allowing to test a theoretical structure such as that proposed in the present study (to assess the 4 factors). This analysis presents some indexes for assessing the goodness of fit (36–40): the chi-square (41, 42) within the incremental fit indices, the comparative fit index (CFI) whose values range from between 0 and 1 and the reference value is 0.90 (43–45) and finally, within parsimony adjustment indices, the error of the root mean square approximation (RMSEA) where the smaller the value, the better the fit, the reference value being 0.05 (46).

Finally, a multigroup analysis was carried out on face-to-face and phone counseling participants to test invariance. Here, a hierarchical procedure must be carried out, beginning with an unconstrained one, and adding constraints successively. The logic of this procedure is to test the factorial homogeneity structure across groups, from a stage where all parameters do not need to be equal to a stage where they have to be. In this way, several authors (47) recommend the invariance analysis on the development of a psychometric test. Moreover, the literature (48) highlights its relevance in the study of parameters from different populations. Therefore, three Models testing configural, metric, scalar invariance were examined across telephone counseling and face-to-face participants.

### Ethics

The studies were approved by the Ethics Committee at the Universidade Federal de Ciências da Saúde de Porto Alegre-UFCSPA, as well as, committees of other centers involved in the research (n.38026014.0.0000.5345).

## Results

The Internal consistency for the whole database in the first sample (*n* = 670) (study I) presented optimal values (α = 0.90 and homogeneity was between 0.17 and 0.5) as well as the subgroups employed in the other two samples (α = 0.91 and homogeneity was between 0.23 and 0.61 for the participants under telemedicine treatment, and an α = 0.90 and homogeneity was between 0.17 and 0.63 for those under face-to-face treatment). An EFA and a CFA were carried out for the participants in the first sample, while a CFA and multigroup analysis were employed in the other two samples.

### Study I- sample 1 (*n* = 670)

The KMO test and Bartlett sphericity test showed optimal values: KMO = 0.932, $X_{\left(\text{df}=630\right)}^{2}$ = 6091.94, *p* < 0.0 < 0.001. The percentage of explained variance was 47.77. Following the Cattell criteria (sedimentation graph self) 7 possible factor solutions were suggested. However, in most of the cases, the percentage of explained variance was very low (approximately 2%). Following both theoretical and empirical criteria, a 4 factor solution was tested. A 3-factor solution was also tested presented less than 3 observations in one factor, for this reason it was rejected. Appendix A contains the item loading for each factor, where a total of 20 items were selected—therefore offering a shorter version than the previous ones. At this stage, items that presented low saturation (<0.40) were removed due to a statistical criterion. Moreover, item 1, as 23 and 26 in factor 2 were not considered because of theoretical criteria (22).

### Study II - CFA analysis: comparison between face-to-face (*n* = 232) and telephone counseling (*n* = 221)

**Figure 1:**
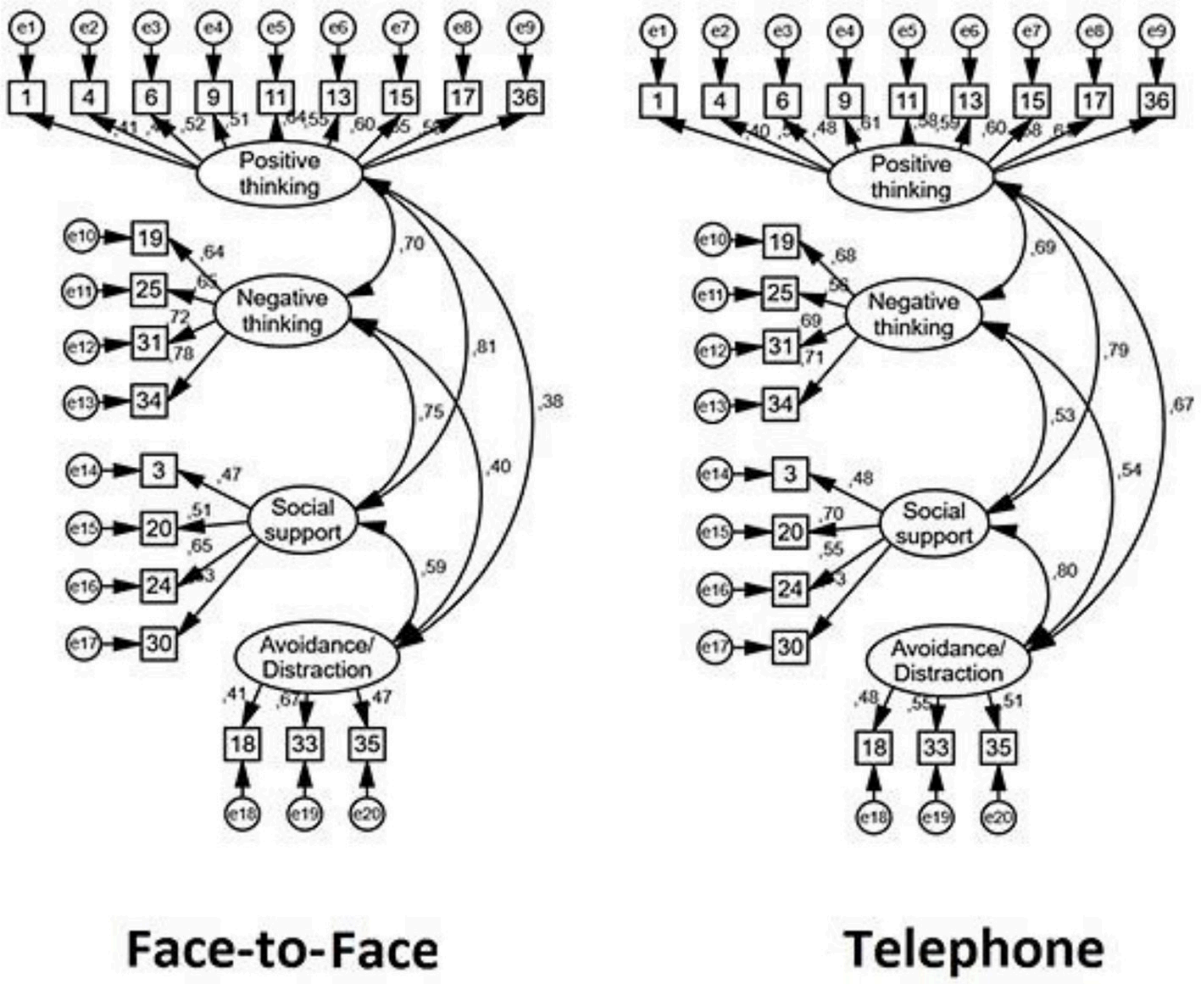
Factor loading for each group (face-to-face and telephone counseling) after the CFA.

In terms of CFA, the new solution was presented across two different samples: Face-to-face and Telephone counseling respondents. More precisely, these indexes were evaluated among the traditional model and the examination developed in 1983, depicting, the current version, a better fit (see Table 1). The CFA employed in the current solution, confirmed an optimal fit for a 4 factors solution in both participant samples. Table 2 depicts the mean score, deviation, error and internal consistency among factors and groups, and Figure 1, the loadings for each group. The variability was slightly higher for telephone in F1 (Positive thinking) and F2 (Negative thinking) in comparison to face-to-face, and the opposite pattern for F3 (Distraction/avoidance) and F4 (Social Support).

**Table 1:** Summary of CFA indexes under assessment.

	**Factorial Structure**	**χ^2^**	**DF**	**χ^2^/DF**	***p***	**CFI**	**RMSEA**
Face-to-Face	(20)	870.06	554	1.57	<0.001	0.857	0.050
	Current reexamination	230.92	165	1.40	0.001	0.934	0.041
Telephone counseling	(20)	778.825	554	1.41	<0.001	0.884	0.043
	Current reexamination	224.54	165	1.36	0.001	0.943	0.041

**Table 2:** Mean score, deviation, error and internal consistency among factors and groups.

**Factor**	**Face-to-Face**			**Telephone**		
	**Mean**	***SD***	**α**	**Mean**	***SD***	**α**
F1: Positive thinking	1.09	0.64	0.797	1.37	0.66	0.795
F2: Negative thinking	1.01	0.70	0.782	1.14	0.71	0.748
F3: Distraction/ Avoidance	1.27	0.80	0.654	1.65	0.80	0.70
F4: Social support	1.85	0.83	0.579	1.97	0.77	0.517

Correlations between IDHEA and CBI were carried out across face-to-face participants (see Table 3), as this is the traditional condition in the field. In terms of convergent validity, the new CBI structure was statistically correlated with the IDHEA questionnaire.

**Table 3:** Correlations between CBI and IDHEA.

		**IDHEA**			**CBI**			
		**F1**	**F2**	**F3**	**F1**	**F2**	**F3**	**F4**
CBI	F1: Positive thinking	0.407^**^	0.187^**^	0.423^**^	1	0.682^**^	0.581^**^	0.245^**^
	F2: Negative thinking	0.345^**^	0.090	0.392^**^	0.682^**^	1	0.549^**^	0.283^**^
	F3: Distraction/Avoidance	0.387^**^	0.112	0.259^**^	0.581^**^	0.549^**^	1	0.338^**^
	F4: Social support	0.203^**^	−0.258^**^	0.107	0.245^**^	0.283^**^	0.338^**^	1

*(Factor 1 - Assertiveness and planning for situations of high risk of consumption of Substances. Factor 2 - Emotional expression of positive feelings for the maintenance of abstinence. Factor 3 - Emotional self-control in adverse situations) and CBI factors (F1-positive thinking. F2-negative thinking. F3-avoidance/distraction and F4- social support), to examine convergent validity*.

* p < 0.0 < 0.005

** *p < 0.0 < 0.001*.

Finally, a multigroup analysis was carried out to determine any significant differences in structural parameters between groups to a metric level. As depicted in Table 4, it is clear that there are no significant changes comparing Model 2 with the less constrained model or baseline.

**Table 4:** Goodness-of-fit statistics for tests of invariance across telephone counseling and face-to-face: a summary.

**Model**	**χ^2^**	**df**	**χ^2^/df**	**CFI**	**RMSEA**	**Δ χ^2^**	**Δ df**	**Decision**
Model 1: Configural invariance (baseline model)	464.848	330	1.397	0.938	0.030	–	–	–
Model 2: Full metric invariance	477.712	345	1.385	0.937	0.029	12.864	15	Accept
Model 3: Full metric and scalar invariance	541.466	365	1.483	0.916	0.033	63.754^*^	20	Reject

* *Remarkable high increment*.

## Discussion

The present study showed that the CBI-brazilian is new version fitted adequately to the original model, even if it suffered changes from its previous versions. Obtaining an adequate instrument and a current evaluation of its structure makes it possible to use it in clinical practice. An accurate assessment and better understanding of coping strategies allow to orient an adaptive coping behavior to cease consumption and maintenance to abstinence. This is not the first time that the questionnaire is reduced, being the previous version of a total of 60 items. With regards to internal consistency, the cronbach's alpha showed optimal coefficients across 3 samples in the studies presented. Moreover, the 4 factor solution presented an optimal fit in terms of CFA. As mentioned before, the extraction of some items was necessary because of a low factor weight of some items. A possible explanation might be directly linked to the current population under study. More precisely, the sample of this study, in comparison to the sample of the original study, present several differences. Here, the participants were not necessarily hospitalized as a common method before. In this way, the application methods have also changed, from the traditional face-to-face administration to the telephone. Nevertheless, it is important to note that the original instrument also presented a 4 factors solution (F1-positive thinking. F2-negative thinking. F3-avoidance/ distraction and F4- social support), the same that was found among the groups of participants. However, items such as number 30 (avoiding places where I drank) seems to fall under the avoidance/ distraction factor. One possible explanation could be that the item follows the first solution proposed for this questionnaire. It is important to bear in mind here that initially this item was stipulated in this factor and moved in the 1983 version by Litman. With regards to item 24 (which moved from the avoidance/distraction factor to social support), we stipulated that something similar happened to the traditional version with item 30.

On the other hand, a similar study performed in Spain also presented subtle alterations in terms of items, keeping the same solution. Apparently, the Spanish adaptation reformulated the “social support” need (27). In this way, the internal consistency found also indicates the weakness of this sub factor. However, its heuristic content in the traditional scale leads to maintain it. This highlight of the reevaluation needs to be addressed for future lines of research.

Despite the fact that CBI is an important inventory for the study of coping with specific reference to alcoholism, its structure has changed over the last 30 years. Therefore, one of the main contributions to the present study is to shed light on their factor structure for alcohol abuse. This issue becomes even more complex when we mention the different subpopulation of alcohol abuse. More precisely, for the Brazilian population, who must overcome geographical barriers, as well as other common obstacles related to social stigmas in this field. The population under study has very specific characteristics. For example, the importance of frequent contacts as this may prevent possible relapses. However, in general, they are people with low income, who tend not to adhere to follow-up. Faced with this reality the telephone help facilitates these contacts and helped to increase this frequency. Or even serve as a kitten in the search for face-to-face help. Therefore, verification that the use of CBI can also be performed with quality via telephone can be an important step in including this instrument in telehealth advice. Therefore, this study also supports its use among different groups to a certain extent. In particular, its invariance across both telehealth and face-to-face on a both configural and a metric level seems to be of interest. In other words, this result suggests that the factor loadings are invariant across populations, offering a questionnaire that psychometrically adapts to both traditional samples and to the new approaches of our era in terms of factor loading. However, one of the limitations of work is that the invariance did not reach the scalar level, also known as strong invariance. As Bollen (44, 45) indicated, this might be indicative of potential measurement bias among items intercepts. Therefore, this suggests differences in the way of responding and rating the items which can be higher or lower among type of administrations. As Gordoni et al. (49) claimed in a study comparing both modalities, the face-to-face way might be superior in terms of item random error. Thus, caution is advised here. The understanding of the operation of evaluation in the healthcare by phone counseling is quite important since it is a trend and has scientific proof of its benefits. In addition, in the locations that exist a large territorial extension and difficulties of investments in health, is a facilitator in the access to these patients.

Further, another important limitation is to be able to test the CBI-20 on larger samples and for longer on follow-up. In this case a psychopathological or psychiatric evaluation would be prudent to conduct because these aspects can influence the results. In this study it was limited to rely only on the patients' reports on the subject. In this way the lack of long follow-ups, might affect the application of the present tool. Therefore, future studies can deepen evaluation the CBI and shed light on new research models on coping assessment (50). More precisely, more research in the validity in predicting relapse and a robust test-retest reliability are imperative in the future: as interventions following a longitudinal process for the test-retest reliability, also described in the previous literature (51–53).

## Author contributions

HC organization and execution of data collection, statistical analysis, writing of the article, review and approved of the final version. CM-T organization of data collection, oriented, conduction and supervision statistical analysis, writing of the article, review and approved of the final version. MCB data collection, statistical analysis, writing of the article, review and approved of the final version. MO organization of data collection, supervision statistical analysis, writing of the article, review and approved of the final version. HB organization of data collection, writing of the article, review and approved of the final version. MF organization of data collection, supervision statistical analysis, writing of the article, review and approved of the final version. All authors have approved the final article.

### Conflict of interest statement

The authors declare that the research was conducted in the absence of any commercial or financial relationships that could be construed as a potential conflict of interest.
