# Supplementary material for: CBI-20: Psychometric Properties for the Coping Behaviors Inventory for Alcohol Abuse in Brazil
Source: Front Psychiatry. 2018 Nov 13;9:585. doi: 10.3389/fpsyt.2018.00585 (PMC6243092; doi:10.3389/fpsyt.2018.00585)
Supplement: Supplementary file 1 [file Data_Sheet_1.PDF]

APPENDIX A: Four-factor solution and item loadings for the CBI-brazilian version (Promax rotation). CBI factors (F1-positive thinking. F2-negative thinking. F3-avoidance/ distraction and F4- social support).

Factor	F1	F2	F3	F4
1. Thinking about how much better off I am without drinking.	**.43**	.06	-.09	.07
2. Telephoning a friend. *	.31	-.09	.21	-.01
3. Keeping in the company of non drinkers.	.28	-.15	-.08	**.45**
4. Thinking positively	**.52**	-.10	-.08	.25
5. Thinking of the mess I've got myself into through drinking. *	.35	.28	.05	-.15
6. Stopping to examine my motives and eliminating the false ones	**.54**	.00	.02	-.06
7. Thinking of the promises I've made to others.*	.35	.14	.19	-.10
8. Staying indoors – hiding.*	-.18	.22	.28	.02
9. Pausing and really thinking the whole alcoholic cycle through.	**.50**	.22	.00	-.15
10. Leaving my money at home.*	-.02	.07	.26	.02
11. Recognizing that life is no bed of roses but drink is not the answer.	**.55**	.14	-.11	.00
12. Going to an A.A. meeting.*	.16	-.00	.19	-.01
13. Knowing that by not drinking I can show my face again without fear of what others will think.	**.47**	.11	.03	.04
14. Cheering myself up by buying myself something special instead.	.43	-.02	.14	.02
15. Facing up to my bad feelings instead of trying to drown them.	**.55**	-.05	.07	-.00
16. Working harder*	.35	-.08	.25	.01
17. Realizing it's just not worth it	**.42**	.15	-.10	.18

18. Waiting it out until everything is shut.	-.04	.07	**.49**	.08
19. Remembering how I've let my friends and family down in the past.	-.04	**.62**	.15	-.05
20. Keeping away from people who drink.	-.04	.04	.09	**.56**
21. Going for a walk.*	.31	-.23	.13	.19
22. Looking on the bright side and trying to stop making excuses for myself.*	.32	.27	-.03	.15
23. Realizing it's affecting my health.	.05	.57	.06	.00
24. Start doing something in the house.	.10	.05	.12	**.40**
25. Considering the effect it will have on my family.	.16	**.57**	-.01	-.01
26. Reminding myself of the good life I can have without drink.	.05	.59	-.13	.10
27. Getting in touch with old drinking friends who are better now*	.23	-.00	.28	.01
28. Making up my mind that I'm going to stop playing games with myself.*	.20	.23	-.06	.35
29. Eating a good meal. *	.22	.06	.21	.06
30. Avoiding places where I drank.	-.19	.05	.16	**.70**
31. Thinking about all the people who have helped me.	-.09	**.54**	.07	.16
32. Saying I am well and wish to stay so.*	.20	.05	-.07	.38
33. Going to sleep.	-.01	-.05	**.47**	.16
34. Remembering how it has affected my family.	-.01	**.72**	.02	-.06
35. Forcing myself to go to work.	.22	-.01	**.42**	-.08
36. Trying to face life instead of avoiding it.	**.43**	.06	.00	.13

***Saturation >.40**
